# Arrears of Contribution

**Published:** 1913-09-13

**Authors:** 


### OUR NATIONAL INSURANCE ACT DEPARTMENT.

### LXVI.—Arrears of Contribution.

Under the National Insurance Act, 1911, if an employee was temporarily unemployed and failed to pay the full contributions as they fell due his rate of sickness benefit derived from his approved society was to be reduced in accordance with a scale depending upon the yearly average of the arrears of contribution. The scheme of the Act had not actually come into operation, so far as employed contributors are concerned, for it was laid down (Section 10, subsection 4) that arrears of contribution were not to count in their case during the first twelve months after the commencement of the Act. The scheme would, however, have come into operation on October 13 next, and the Insurance Commissioners had issued regulations for the purpose of determining the manner of calculating the average arrears.

The whole question has been substantially altered by the Amendment Act, and in our opinion the new scheme is a vast improvement upon the old. The calculation of the average arrears, even in the most favourable circumstances, would have been a tedious process, and would have added immensely to the administrative difficulties imposed upon the approved societies in paying benefits. It is also likely that dissatisfaction would have been expressed by members of approved societies no matter what method of calculation had been adopted. Furthermore, the Commissioners stated that while the Act enforced the reduction of benefits in proportion to the average arrears it was impossible for them to sanction the issue of a half-yearly contribution card in place of the present quarterly cards. The demand of the approved societies for half-yearly insurance cards as a means of reducing administrative expense was so reasonable, and so unanimous from all classes of approved societies, that it became necessary to take some steps to make the substitution possible.

Thus the case for the amendment of the original Act in regard to arrears of contribution was abundantly proved.

### Arrears of Employers' Contribution Excused.

The provisions of the Amendment Act in this matter are as yet not generally appreciated, because they have not yet come into operation; but it is important that all members of approved societies should realise as soon as possible the nature of the alterations that have been made. In the first place the Amendment Bill as introduced proposed to make it obligatory on the approved societies to accept the employed person's own contribution (4d. men and 3d. women), and to excuse the payment of the employer's share of the contribution when the insured person desired to pay up the arrears for any period during which he was unemployed. Under the original Act the approved societies were allowed to make this concession if they chose to do so, but there was no obligation imposed upon them.

The proposal received legislative sanction, and is now embodied in Section 7 of the Amendment Act.

This is a substantial concession to employed contributors, and removes the reproach that had been levelled against the principal Act, namely, that insured persons had to pay more when unemployed—or to make it up afterwards by paying arrears—than when in regular employment, or else run the risk of reduction of or disqualification from benefit.

The interests of the approved societies—that is to say, the interests of the insured persons as a whole—are safeguarded by the provision that if the average arrears in the case of any society exceed three weekly contributions per member in the course of a year the loss shall be made good to the society out of the sinking fund retained by the Insurance Commissioners for reducing the initial deficiency in the reserves.

### Scheme for Reduced Benefits Repealed.

The remaining provision of the Amendment Act (Section 8) dealing with the question of arrears did not form part of the original amending Bill, but was introduced during the Committee stage.

It is perhaps the most drastic of the alterations made by the Amendment Act of the original structure of the National Insurance scheme, as it virtually supersedes the whole plan for the reduction of benefits on account of arrears of contribution as set out in the principal Act. The original scheme required a very long section of the Act and the whole of the fifth schedule to itself. The new proposals are much less elaborately set forth, though it remains to be seen whether the regulations of the Insurance Commissioners which are to govern their application will follow in the same direction.

Apart from the subsection of the principal Act, which provides for the exceptions from the operation of its provisions, it is now laid down that the whole of these provisions shall cease to have effect.

The exceptions are important. No account is to be taken of arrears when the insured person has been in receipt of sickness or disablement benefit, or would have been in receipt of such benefit if not disqualified by other provisions of the Act. In the same way arrears are not to count for two weeks before and for four weeks after confinement when a woman is entitled to maternity benefit from her own insurance, nor when the maternity benefit is payable in respect of a posthumous child during the period subsequent to the father's death. Any arrears falling due during the first twelve months after the commencement of the Act are also to be excused.

These were the original exceptions and they still hold under the new scheme of the Amendment Act.

Subject to this proviso the whole of the original scheme has been repealed, and in its place it is now provided (Section 8) that "insured persons who are in arrear shall be liable to such reduction, postponement, or suspension of benefits as may be prescribed so, however, that any such reduction, postponement, or suspension of benefit shall be approximately equivalent to the value of the loss occasioned by the failure to pay the contributions in arrear . . . and the regulations of the Insurance Commissioners may prescribe the time within which, and the conditions under which, arrears may be paid up."

Insurance Commissioners to Issue Regulations.

At first sight this appears very vague and indefinite, and the point that will at once command attention is the fact that the scheme is to be embodied in regulations formulated by the Insurance Commissioners. It is perhaps as well that in a matter of this sort the precise form should be left for the Insurance Commissioners to settle, for the details, to be satisfactory, must admit of easy application, and it is difficult to draw up a scheme so perfect in every particular that it will admit of no modification. If the scheme had been definitely laid down in the Act no alteration, however urgent, that might have been demanded as the result of practical experience of its working could have been made except by means of a further enactment. Under the present arrangement the Insurance Commissioners may alter their regulations whenever a clear case can be shown that they should be so altered. This was probably one of the reasons that prompted the Government to accept an amendment giving the Commissioners the power to issue regulations to meet the necessities of the case. There was probably another reason also, for it would seem that a completely satisfactory scheme had not been devised when the Bill was before Committee.

The broad outlines of the suggested scheme had, however, been drawn up, and these are set forth in a "Note on Clause 8 (Calculation of Arrears)."

Outlines of the New Scheme.

For the moment we only have the suggested scheme. Within the next few weeks the actual regulations will be published. It may, however, be taken for granted that the regulations will follow fairly closely the principles that have been laid down. It was deemed essential to preserve to the insured persons all the rights accruing from the regular payment of contributions accorded to them by the Act. For this reason it is suggested that when forty-eight weekly contributions have been paid—or credited as paid, as, for instance, when the insured person has been in receipt of sickness benefit—this should be considered as the standard number of contributions and should entitle the person to full benefits.

It was also deemed to be necessary as far as possible to ensure equity of treatment between persons of different age. This was not attempted in the scheme of the principal Act, the same scale of reductions applying at all ages. To meet this requirement the scale of reductions will be graded with regard to the age attained at the time the member falls into arrears.

The third essential characteristic of a satisfactory scheme was deemed to be simplicity of administration, and to meet this it is suggested that the reduction of benefit should be made to operate for single years, and thus obviate the necessity for the calculation of an average of arrears of contribution.

As forty-eight weekly contributions is to be considered as the standard entitling the member to full benefits, any excess above this number paid in any year are to be carried forward to his credit in future years. Thus, if in one year fifty-two contributions are paid, in the following year it would be possible for the member to fall into arrear to the extent of eight weeks, four of which are carried forward as a credit from the former year, without affecting his right to full benefits. Every employed contributor entering into insurance during the first year after the commencement of the Act will be given a credit of either four, three, two, or one contributions to carry forward to subsequent years according as he entered insurance in the first, second, third, or fourth quarter.

It is suggested to make the penalty in the case of a man who is under forty-five years of age a reduction of sickness benefit of 6d. a week for each contribution in arrear. For ages forty-five to fifty the suggested reduction is 5d., between fifty and fifty-five it is 4d., and above fifty-five it is 3d. Disablement benefit would be reduced at half these rates, and lower rates would apply to women than to men. It is also suggested that a maximum limit should be placed on the reductions, so that if this maximum be exceeded the member would for the time being be suspended altogether from benefit.

A special feature of the proposal is that at the end of the year during which the rate of sickness benefit is reduced the debt will have been cleared off, whether the member has himself been ill or not. The reduction in the rate of benefit corresponds as nearly as may be to the average expectation of sickness, and it is this average that has been looked to rather than the sickness of an individual insured person. If further arrears have accumulated during the year in which the rate of benefit has been reduced the member would be subject to the reduced rate of benefit applicable to those fresh arrears, but all old scores would be paid off.

Thus a person who has a spell of bad fortune and is thrown out of employment for a considerable time in one year will not carry the weight of that disadvantage in the form of a reduction of his rate of sickness benefit for the remainder of his insured lifetime as he might have done under the old scheme.

Approved Societies and Half-Yearly Cards.

The approved societies, we understand, welcome the scheme not only on its own merits as a simple method of dealing with arrears, but also because it makes it possible for half-yearly insurance cards to be introduced. Is it too late even now, although the sixth quarter's cards have been printed, to make the arrangements for half-yearly cards to commence in October?

[A letter on "Panel Doctors and Prescription Writing" which raises several interesting points appears in our correspondence columns this week.—Ed. The Hospital.]

